# Report on a conference analyzing the role of cerebrospinal fluid prophylaxis for brain tumors

**DOI:** 10.1186/1743-8454-5-6

**Published:** 2008-03-26

**Authors:** Michael Glantz, Conrad Johanson

**Affiliations:** 1 Huntsman Cancer Institute, University of Utah School of Medicine, 2000 Circle of Hope, Salt Lake City, Utah 84112-5550, USA; 2 Department of Clinical Neurosciences, Warren Alpert Medical School of Brown University, 593 Eddy Street, Providence, RI 02903, USA

## Abstract

This is a report of a meeting sponsored by MundiPharma International to identify ways to exploit the cerebrospinal fluid system pharmacologically, for more effective management and prevention of primary and metastatic CNS tumors.

## Background

Thirty investigators from numerous cancer research centers in the United States and Europe were convened by conference organizer, Michael Glantz (University of Utah School of Medicine – Huntsman Cancer Institute), to brainstorm the understanding of novel mechanisms, and the development of new paradigms involving the cerebrospinal fluid (CSF) system, for thwarting the spread and improving the treatment of nervous system cancers. This retreat brought oncologists and clinical neuroscientists to St. Thomas, Virgin Islands on February 22–24, 2007. The conference, entitled '*The Science of Prevention: New Insights into the Importance of Intra-CSF Prophylaxis*', was organized around several provocative theses to stimulate discussion of novel ways to treat, and even prevent, brain and meningeal tumors.

## Discussion

Pathophysiogically and pharmacologically, the CSF is an important circulatory system in the brain and spinal cord that, on the one hand disseminates malignant cells, but on the other hand might be harnessed to distribute corrective chemotherapeutic drugs. As knowledge of the CSF system increases, so do the opportunities for managing brain cancer. Successful CSF prophylaxis for medulloblastoma and acute leukemia could well serve as a paradigm for more effective treatment of lymphomas. Conference introductory keynotes by Ching-Hon Pui (St. Jude Children's Research Hospital) and Eckhard Thiel (Charite University Medicine Berlin) provided historical perspective and state-of-the-art reviews on acute leukemia and non-Hodgkin's lymphoma. Michael Glantz then addressed the feasibility of a randomized, controlled trial of CSF prophylaxis in patients with high-risk non-Hodgkin's lymphoma. The treatment-refractory glioblastoma multiforme, in dire need of a new therapeutic model, was addressed by Lawrence Recht (Stanford University Medical School) who analyzed gliomagenesis in the context of growth factor effects on cancer stem cells in the subventricular zone. Utilizing new basic science information, Glantz then proposed a phase II trial incorporating intra-CSF chemotherapy into the initial treatment of patients with newly diagnosed glioblastoma multiforme.

Three decades of experience with neoplastic meningitis (NM) has afforded many clinical insights on treatment, but cures remain exceedingly rare, and controversy persists regarding the most basic tenets. Marc Chamberlain (Fred Hutchinson Cancer Center) reviewed prognostic variables within subgroups of patients with solid tumor neoplastic meningitis, and urged tumor-specific strategies for CSF-directed therapy. However, the whole concept of intrathecal chemotherapy as a treatment for solid tumor NM was questioned by Willem Boogerd (Netherlands Cancer Institute) who emphasized the urgent need for a randomized clinical trial to address this issue, and presented such a proposal on behalf of the European Organisation for Research and Treatment of Cancer (EORTC). The rationale and techniques for treating malignant cells in CSF were discussed by William Shapiro (Barrow Neurological Institute), while the expectations for significantly improving survival in solid-tumor patients were outlined by Stuart Grossman (Johns Hopkins Medical Institutions). Conrad Johanson (Brown University – Warren Alpert Medical School) introduced the potential role of pharmacologically modifying choroid plexus function to treat or prevent cancer spread along the ventriculo-subarachnoid CSF system. Recognizing the limitations of CSF cytology and magnetic resonance scanning, Glantz outlined the need for more sensitive techniques to diagnose CSF malignancies and evaluate treatment outcomes. The importance of novel markers for diagnosing and monitoring responses in NM and lymphomatous meningitis was extensively treated by Morris Groves (M.D. Anderson Cancer Center) and Wyndham Wilson (National Cancer Institute).

The final session of the conference was both practical and visionary. Clinical trials, both newly completed and projected, were discussed. New insights for protecting against brain metastases (including lung and breast cancer) were delineated by Wallace Akerley (University of Utah School of Medicine – Huntsman Cancer Institute), Frankie Holmes (Baylor College of Medicine) and Ricardo Soffietti (University of Turin). Gudrun Fleischhack (University Children's Hospital, Bonn) introduced opportunities for pediatric primary brain tumor initiatives, while Ulrich Herrlinger (University of Bonn) discussed primary central nervous system lymphoma. Randy Jensen (University of Utah School of Medicine – Huntsman Cancer Institute) reviewed the evidence in favor of post-neurosurgical prophylaxis. Finally, Jennifer Smith (Geron Corp) treated statistical issues in designing critical trials, including identifying predictive and prognostic markers. She also offered advice on clinical trial proposals developed during the conference.

## Conclusion

This *Science of Prevention *conference was the first of its kind to focus on the CSF as a medium around which to marshall research and clinical strategies for preventing and treating brain cancer. Brain tumor development or spread, from both the pathologic and the pharmacologic point of view, is critically dependent upon functional relationships between CSF dynamics and brain metabolism. Considerably more information is needed about CSF-bordering cells (e.g., choroid plexus, ependyma and the arachnoid) in order to more effectively thwart dissemination of cancer cells throughout the CSF and interstitial fluid compartments. Also, analysis of primary brain tumor development in the subventricular zone deserves more attention as a site for modulating (by growth factors and antagonists) stem cell oncogenicity. The efficacy of intrathecal *versus *combined intrathecal and systemic administration of chemotherapeutic drugs also merits more attention. Clearly new CSF markers and diagnostic techniques are essential for improving disease identification and monitoring response to therapy in a class of diseases which continues to challenge oncologists, neurologists, neurosurgeons, basic scientists, and, most especially, patients. As a result of this conference, the rationale for expanding the intra-CSF prophylaxis approach to brain and meningeal tumors has now been more sharply defined.

## Competing interests

The author(s) declare that they have no competing interests.

## Authors' contributions

Both authors contributed equally to this report and have read and approved the final version of the manuscript.

